# The Effect of Chlorhexidine Mouthwashes on the Microbiota Associated with Peri-Implantitis Lesions: A Pilot Study

**DOI:** 10.3390/antibiotics14101032

**Published:** 2025-10-15

**Authors:** Félix Pose-Otero, Alexandre Arredondo, Ana Parga, Andrea Muras, Mercedes Gallas, Paz Otero-Casal, José Manuel Pose-Rodríguez, Ana Otero

**Affiliations:** 1 Departamento de Cirurxía e Especialidade Médico-Cirúrxica, Facultade de Medicina e Odontoloxía, Universidade de Santiago de Compostela, 15705 Santiago de Compostela, Spain; felixramon.pose@rai.usc.es (F.P.-O.); mercedes.gallas.torreira@usc.es (M.G.); paz.otero.casal@sergas.es (P.O.-C.); josemanuel.pose@usc.gal (J.M.P.-R.); 2 Department of Microbiology, Dentaid Research Center, 08290 Cerdanyola del Vallès, Spain; alex.arredondo@dentaid.es; 3 Department of Microbiology and Parasitology, Faculty of Biology, Universidade de Santiago de Compostela, 15705 Santiago de Compostela, Spain; ana.parga.martinez@usc.es (A.P.); andrea.muras.mora@sergas.es (A.M.); 4 Aquatic One Health Research Center (ARCUS), Universidade de Santiago de Compostela, 15705 Santiago de Compostela, Spain; 5 Unit of Oral Health, C.S. Santa Comba-Negreira, Servicio Galego de Saúde (SERGAS), 15830 Negreira, Spain

**Keywords:** peri-implantitis, oral microbiota, oral biofilm, chlorhexidine, 16S rRNA gene amplicon sequencing, Illumina MiSeq

## Abstract

**Background/Objectives:** Chlorhexidine (CHX)-based mouthwashes are the most commonly used chemical methods as adjuvants for the treatment of peri-implant diseases, but there is a lack of information on their effect on the peri-implant microbiota. The objective of this study was to evaluate the impact of short-time (15 days) self-administered 0.05% and 0.12% commercial chlorhexidine mouthwashes on the peri-implant pocket microbiota of patients with peri-implantitis. **Methods:** In this pilot study, we analyzed the microbial composition of peri-implant lesions in 22 patients before and after a 15-day regimen of thrice-daily use of two commercial chlorhexidine mouthwashes containing 0.05% (*n* = 11) and 0.12% chlorhexidine (*n* = 11). Microbial samples were collected using paper points, and the taxonomic composition was determined through sequencing of 16S rRNA gene amplicons using Illumina MiSeq. **Results:** Although individual responses to chlorhexidine mouthwash treatment varied significantly, neither concentration produced a statistically significant change in the microbial diversity associated with peri-implantitis, suggesting limited treatment penetration into peri-implant lesions. Similarly, changes in the abundance of specific odontopathogens were not statistically significant. **Conclusions:** We found no significant differences in the effect of mouthwashes with different chlorhexidine concentrations on the peri-implant microbiota in short-period applications. Even though more extensive studies are required, the observed patient-dependent outcomes of both chlorhexidine mouthwashes on the peri-implant microbiota and their limited effect in controlling the abundance of oral pathogens indicate that prescription of chlorhexidine mouthwashes for the treatment of peri-implantitis should be carried out with caution. Novel presentations of chlorhexidine with better penetration capacities should be developed, as they could offer enhanced benefits in managing peri-implant diseases.

## 1. Introduction

The use of implants for dental restoration has become a common practice in oral healthcare to replace missing teeth. Despite the improvements in oral health and life quality derived from the development of dental implant technologies, the lifespan of these components can be shortened when complications occur, producing a huge impact, both economically and in terms of the life quality of the patients [1]. In this sense, the survival rate of dental implants depends on the environmental conditions of the oral cavity, with infection being the most frequent cause of dental implant failure [2,3].

The peri-implant diseases include peri-implant mucositis (PIM), a reversible inflammation of the soft tissue around dental implants, and peri-implantitis (PI), an irreversible inflammation accompanied by bone loss [3,4], even though the borderline between these two stages can be blurry [5]. Although knowledge about the microbial etiology of peri-implant diseases is still limited [6,7,8,9], they have been assimilated into standard periodontal processes since they present an increase in the number of Gram-negative proteolytic anaerobic microorganisms in comparison to implants surrounded by healthy tissue [10,11]. Therefore, PIM and PI are traditionally considered analogs of gingivitis and periodontitis. However, the etiological relationship between periodontitis and PI disease is unclear, as the results reported in the literature on this topic are contradictory [12,13,14]. The PI disease seems more related to the host’s response to the oral bacterial community than to the presence of periodontopathogens per se [7]. This immune system overreaction could be promoted by host factors, material, and physicochemical properties of the implant, and specific microbial species [15]. Although there seems to be a common “core microbiota” common to periodontitis and PI [8,9,16,17], some species have been specifically associated with one or the other [12]. On the contrary, other studies report that the microbial diversity of PI and periodontitis lesions is relatively similar, suggesting that the same pathogens may be causing both diseases [13,14].

Mechanical debridement constitutes the standard and most effective non-surgical treatment method applied for the treatment of PI. Several novel adjunctive treatments have been explored, including the administration of systemic/local antimicrobials and probiotics [18]. Nevertheless, among the chemical methods used as adjuvants for the treatment of peri-implant diseases, treatment with chlorhexidine (CHX)-based mouthwashes is still the most commonly applied approach since it is considered the most effective compound that reduces oral biofilm formation, gingival inflammation, and bleeding [19,20,21,22]. The use of CHX in combination with the mechanical treatment of PIM has been associated with a reduction in peri-implant inflammatory parameters [22,23,24]; in fact, the prolonged (6 months) use of combined CHX treatments (0.12% CHX mouthwash, subgingival CHX irrigation, and 1% CHX gels) caused a significant decrease in bleeding on probing (BOP) [25]. On the contrary, a meta-analysis concluded that the adjunctive therapy with CHX may not improve outcomes with non-surgical management of PMI, and no clear conclusion could be drawn for the treatment of PI [26], and CHX did not demonstrate a significant effect in the mean counts of *P. gingivalis* after non-surgical periodontal therapy [27]. Moreover, even though the long-term use of CHX in these pathologies is very common, it should be noted that this antimicrobial compound presents undesired, and in most cases, underestimated, negative effects in the long term. For example, repeated CHX treatment can cause an increase in minimum inhibitory concentration (MIC) against antiseptics [28,29]. CHX also alters the surface topography of dental implants and has cytotoxic effects on osteoblasts, therefore potentially hindering osseointegration [30]. Damage to leukocytes and other oral mucosal cells has also been described [31]. Furthermore, the appearance of cross-adaptation to other antibiotics has also been reported [28]. Other side effects of CHX include soreness in the tongue and mouth, interference with taste, or teeth staining [32,33,34].

The application of new techniques related to microbial ecology is needed to evaluate the anti-infectious approaches used in the prevention and treatment of oral diseases such as periodontitis and PI, which depend on the microbial profile of the patients and their response to treatment. Most studies evaluating the effect of CHX on microbial composition use in vitro models based on a limited number of oral strains [35,36,37,38], and only a few were performed using next-generation sequencing (NGS) technologies on in vitro microcosm biofilms [39,40] and in vivo samples [41,42,43,44]. It has been described that the broad-range antimicrobial activity of CHX may cause a shift towards lower bacterial diversity in oral biofilms [41,43,44], and even though this shift could favour the resolution of dysbiosis in periodontal diseases [45], more evidence is required to assess the long-term effect of these changes. This shift also affects bacterial metabolic activities, including changes in pH-buffering capacity and salivary concentrations of lactate, nitrate, and nitrite [40,42,46], reducing the protective barrier against pathogens provided by the commensal microbiota [47]. Since CHX can decrease the abundance of nitrate-reducing oral strains, its prolonged use may increase blood pressure [48]. Moreover, several reports point to a detrimental effect of CHX on the recovery to homeostasis of the oral ecosystem, even promoting dysbiosis [40,46].

The available studies on the effect of CHX on oral microbiota composition focus on periodontitis lesions, and thus, these results cannot be directly translated to PI due to intrinsic differences in the anatomy of both types of lesions that affect the penetration of the antiseptic. A better understanding of the response of PI-associated microbiota to CHX exposition could guide the development of novel strategies to control PI progression. Therefore, the objective of this study was to evaluate the impact of short-term (15 days) self-administered commercial chlorhexidine mouthwashes on the peri-implant pocket microbiota of patients with peri-implantitis. This work used NGS techniques to compare the bacterial communities’ composition obtained directly from PI lesions from the same patients before and after a 15-day treatment with commercial products containing 0.05% or 0.12% CHX.

## 2. Results

### 2.1. Subject Recruitment and Characteristics

Twenty-two patients with PI were recruited from the University Dental Clinic of the Faculty of Medicine and Dentistry in the Universidade de Santiago de Compostela (Spain). Patients in both groups had an average age of 65 years, with an equal percentage of males/females (Table 1). Other clinical parameters, such as pocket depth, bleeding on probing, or duration of PI symptoms, implant type, and placement date, were recorded. Risk factors such as smoking habits, medical conditions/treatments, etc., were also recorded (Supplementary Tables S1 and S2). Non-smokers represented 91% of the patients in both treatment groups. All patients received their implants at least 2 years before the study and presented symptoms of PI for more than one month. None of the patients were treated with CHX or antibiotics for fifteen days or three months, respectively, before the sampling procedure. All recruited patients met the study requirements, which was facilitated by the short period of prescription (15 days).

### 2.2. Effect of CHX Treatment on Microbial Diversity

**Figure 1:**
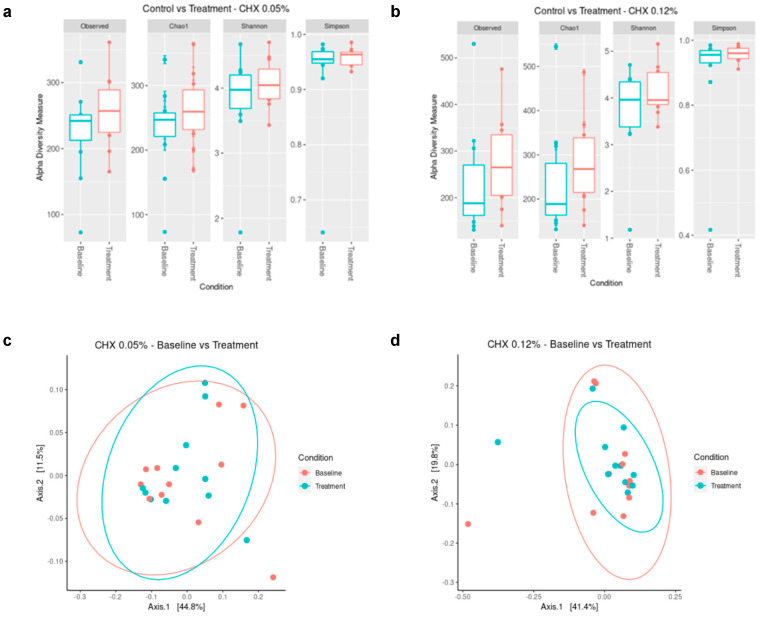
Richness and diversity indices (**a**,**b**) and Principal Coordinates Analysis of weighted Unifrac plots (**c**,**d**) of the microbiota structure of subgingival peri-implantitis (PI) samples obtained before (baseline) and after receiving the different chlorhexidine (CHX) treatments for 15 days (*n* = 11). (**a**,**c**): Baseline vs. Post-treatment with 0.05% CHX (*n* = 11); (**b**,**d**): Baseline vs. Post-treatment with 0.12% CHX (*n* = 11).

The number of genera found in PI pocket samples was 35–76 for the 0.05% CHX group, 39–159 for the 0.12% CHX group at the baseline, and 55–80 and 42–78 for the corresponding groups after the treatments. Overall, alpha diversity values were higher in the post-treatment samples compared to the baseline (Figure 1a,b), even though these differences were not statistically significant (Supplementary Table S2). Similarly, no significant differences were observed between the microbial composition of the populations as assessed by weighted Unifrac analysis (Figure 1c,d and Supplementary Table S2). Even though differences have been described in the implant-associated microbiome of smokers and non-smokers [49], in our case, smoker samples clustered with the non-smoker samples, hence justifying the inclusion of smokers in follow-up analyses.

**Figure 2:**
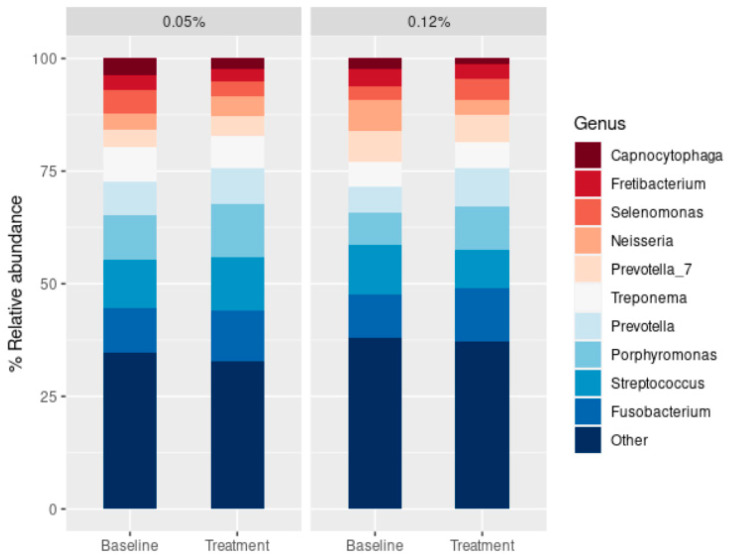
Relative abundance of the 10 most abundant genera observed in the subgingival peri-implantitis (PI) samples obtained before (“baseline”) and after (“treatment”) receiving the 0.05% chlorhexidine (CHX) and 0.12% CHX for 15 days. The category “other” accounts for the sum of the remaining genera identified in the samples.

Both baseline and post-treatment samples were dominated by members of the genera *Fusobacterium*, *Streptococcus*, *Porphyromonas*, *Prevotella*, and *Treponema*, with the smaller representations of *Selenomonas*, *Fretibacterium*, and *Capnocytophaga* (Figure 2). The 10 most abundant genera in the samples accounted for roughly 62% of all the genera identified in all the conditions studied. The relative abundance of the main genera was very similar between CHX treatments and compared to the initial samples (Figure 2). When the relative abundance of certain bacterial genera was assessed after CHX treatment, significant differences were only found for the genus *Jonquetella* in the 0.05% CHX treatment and *Cardiobacterium*, *Actinobacillus*, *Blautia*, *Suttonella*, and members of the *Eubacterium yurii* group for the 0.12% CHX treatment (Supplementary Materials Table S3).

### 2.3. Effect of the CHX Treatment on the Abundance of Main Periodontopathogens

**Figure 3:**
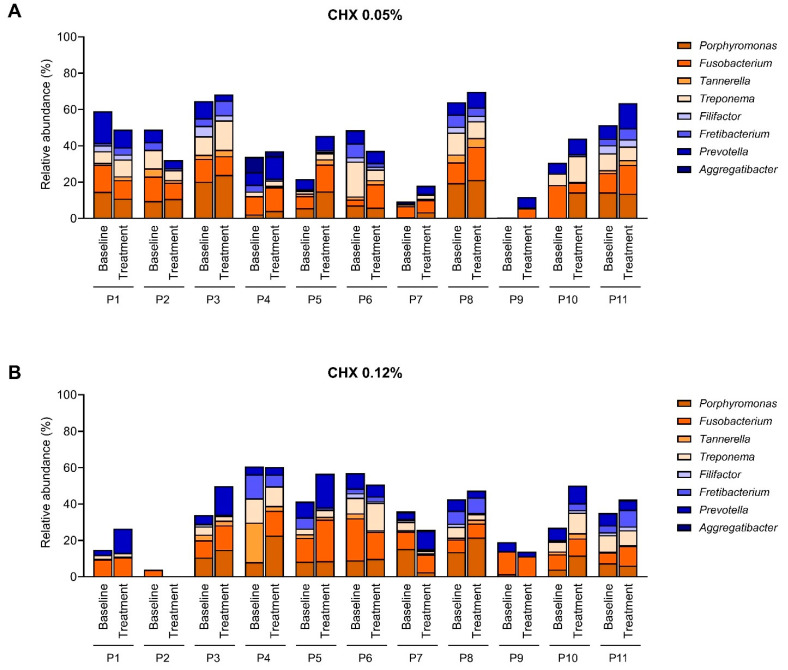
Relative abundance of 8 selected periodontopathogens (*Porphyromonas*, *Fusobacterium*, *Tannerella*, *Treponema*, *Filifactor*, *Fretibacterium*, *Prevotella*, and *Aggregatibacter*) in the subgingival peri-implantitis samples before (“baseline”) and after (“treatment”) 15 days of treatment with 0.05% chlorhexidine (CHX) (**A**) (*n* = 11) and 0.12% CHX (**B**) (*n* = 11).

Despite this general homogeneity in the relative abundance of the main genera identified in the samples (Figure 2), the abundance of pathogens traditionally associated with PI varied greatly among patients (Figure 3). Members of the genera *Porphyromonas*, *Fusobacterium*, *Treponema*, and *Prevotella* were dominant in almost all subjects, with relative abundances ranging from 0 to 24%. Several patients presented with completely different baseline profiles. For patients P7 and P9 from the 0.05% CHX group and patient P2 from the 0.12% CHX group, the abundance of oral pathogens at baseline was very low, reaching less than 10% of total abundance. The different initial composition of patient P9 from the 0.05% CHX group could be explained by his epilepsy (Supplementary Material Table S4) because individuals with this condition usually present poor oral hygiene [50]. No putative causative agent could be identified in the clinical history of patients P7 from the 0.05% CHX group and P2 from the 0.12% CHX group to explain the low abundance of pathogens in their lesions.

Although the effect of CHX on the abundance of these oral pathogens displayed a high inter-individual variation, exposition to CHX appeared to increase the relative abundance of these genera in most patients regardless of the CHX concentration in the mouthwash, even though such differences were not statistically significant (Figure 3).

## 3. Discussion

The use of mouthwashes containing CHX is commonly prescribed for the ambulatory treatment of periodontal diseases such as periodontitis or PI due to the capability of this compound to reduce oral biofilm formation, gingival inflammation, and bleeding, mainly in long-term applications [19,20,22,24,25,41,51]. Nevertheless, the broad-range activity of CHX has raised the question of the impact of CHX on microbial community composition [29,46,47]. Despite the relevance of this question, the effect of CHX has been mainly tested in vitro on different biofilm models [38,39,40,52], and only a few NGS-based in vivo studies have been performed, analyzing the effect of CHX treatment on experimental gingivitis [41], healthy patients [42], or patients with PIM [24,25,44]. Considering the undesirable side effects of CHX, a deeper understanding of PI-associated microbiota responses to CHX mouthwashes is needed. Such knowledge could allow the selection of patient-tailored therapies and the development of novel strategies to control the progression of PI. This pilot study examined the impact of two self-administered commercial mouthwashes containing 0.05% and 0.12% CHX over 15 days on the oral microbial composition within peri-implant pockets of patients with PI.

The results showed that the bacterial composition of the samples and the effect of treatment with CHX (0.05% or 0.12%) were strongly patient-dependent. Several studies point to a higher microbial diversity of PIM and PI sites in comparison with healthy implant sites [8,9]. In our case, average alpha diversity values were higher in the post-treatment samples compared to the baseline of PI sites (Figure 1a,b), which may indicate an increase in the associated dysbiosis. In any case, overall, these differences were not statistically significant for any of the CHX treatments due to the large differences among patients (Figure 1, Supplementary Tables S1 and S2) and indicating that the study of a larger number of patients is required in order to draw solid conclusions. Moreover, no differences were found between samples’ diversity and microbial composition from the 0.05% CHX and 0.12% CHX treatment groups (Supplementary Tables S1 and S2). Previous in vitro studies could not find any correlation between chlorhexidine concentration in commercial mouthwashes and the observed changes in multispecies oral biofilms [38]. A previous study pointed to a negligible effect of the dead cells on the evaluation of microbial diversity in dental plaque after mouthwash treatment [53] and crown surfaces were cleaned before sampling to avoid contamination with supragingival plaque. Therefore, the observed absence of significant differences among treatments points to a lack of efficacy of the CHX mouthwashes on the microbiota associated with PI lesions. It should also be noted that both commercial mouthwashes evaluated contain cetylpyridinium chloride at a concentration of 0.05%, which could have affected the observed results. In any case, the absence of response to the treatment in both groups excludes the effect of this compound. Moreover, no correlation was observed between the presence of cetylpyridinium chloride in commercial mouthwashes and the composition of in vitro developed multispecies oral communities [38]. Previous in vivo studies have reported significantly lower microbial diversity after using CHX in saliva and subgingival samples from healthy subjects or subjects with experimentally induced gingivitis [41,42] and in patients with PIM [44], although these studies used higher concentrations of CHX (0.2% CHX). In vitro studies also describe a strong effect of CHX on the number of viable bacterial cells recovered [54] and microbial diversity [39,40,52]. Here, the limited effect reported for the CHX treatment on the microbiota associated with PI lesions may stem from a poor penetration of the mouthwash in the PI pocket, as previously reported in a multispecies clinical biofilm model [55] and points to the need for developing novel presentations and modes of application of CHX to guarantee its effective penetration into the PI pocket.

The genera *Fusobacterium*, *Streptococcus*, *Porphyromonas*, *Prevotella*, and *Treponema* dominated both baseline and post-treatment samples, with no statistically significant differences among them (Figure 2). Contrarily, previous studies carried out with saliva samples from healthy donors reported an increase in the relative abundance of members of the Bacillota phylum after CHX treatment [42], increasing the salivary concentration of lactate and reducing its buffering capacity, therefore driving to a higher risk of oral disease. Moreover, CHX can decrease the abundance of nitrate-reducing oral strains [42,46], which may increase blood pressure [42,43,48]. Since nitrite has been shown to have an inhibitory effect on the growth of periodontopathogens [42,56,57], the use of CHX for the control of PI must be carried out under the surveillance of oral ecological conditions to avoid driving the oral microbiota towards a dysbiotic state in the long term.

Here, a high abundance of periodontopathogens previously associated with PI [6,8,9,58], including *Aggregatibacter*, *Fusobacterium*, *Porphyromonas*, *Tannerella*, *Prevotella*, and *Treponema*, was observed, even though differences in their relative abundance were observed among patients (Figure 3). None of these genera were significantly affected by the CHX treatments, while those significantly affected by the CHX treatments were not specifically associated with the development of PI (Supplementary Materials Table S3). Interestingly, the summation of the relative abundance of these oral pathogens was slightly increased in the 0.05 CHX group, from 39% in the baseline to 41% in the post-treatment samples, and also in the 0.12% CHX group, from 34% in the baseline to 38% in the post-treatment condition, although these differences were not statistically significant. A previous study reported that daily exposure to 0.12% CHX can promote the presence of periodontopathogens, aggravating dysbiosis, and showing a repeated pattern of inactivation and rapid regrowth of in vitro oral biofilms [40]. It should also be noted that periodontopathogens usually live in the deeper layers of the oral biofilm [40,59] and, therefore, are even more protected from the action of antiseptics than bacteria that occupy more superficial areas of the biofilm. Additionally, repeated treatment with CHX has been reported to increase the MIC of antiseptics and to foster the appearance of cross-adaptation to other antibiotics, accompanied by changes in cell surface hydrophobicity and protein expression [28,29,60]. In this sense, persistent populations have been reported in vitro after treating oral biofilms with 2% CHX [61]. Therefore, the use of the lowest CHX concentration would be recommended.

Although CHX has been proven effective in controlling the biofilm formation and clinical symptoms of gingivitis, its efficacy in managing PI requires a deeper knowledge of the effect on the microbiota associated with the PI lesion [46]. In view of the observed changes in alpha diversity, the treatment with CHX to manage PI may need close surveillance to guarantee that the oral microbiota is not shifted to a dysbiotic state [28,40]. The evidence presented herein on the limited efficacy of CHX formulations to modify PI-associated microbiota is short-period applications, and the complications derived from the long-term use of CHX mouthwashes point to the need for alternative control approaches that promote the proliferation of health-associated strains [62,63] or interfere with the virulence traits [64,65], even though the study of the response of a larger number of patients would be required to ascertain the results obtained in this pilot study.

## 4. Study Limitations

This pilot study has several important limitations that should be considered when interpreting the results:

1. Only 22 patients were included, equally divided between two treatment groups. This limited cohort reduces statistical power and makes it difficult to detect subtle but potentially relevant differences in microbiota composition. Moreover, the microbial response to CHX was strongly patient-dependent, with marked differences in baseline microbiota composition and treatment outcomes. This variability complicates the interpretation of group-level effects. Larger-scale studies are necessary to confirm these preliminary findings.
2. The intervention lasted only 15 days; therefore, the results cannot be extrapolated to the longer durations of treatment often prescribed in clinical practice. While long-term treatment may have other undesirable effects, short-term exposure may underestimate both beneficial and adverse effects on peri-implant microbiota.
3. The commercial mouthwashes used contained not only CHX but also cetylpyridinium chloride. The combined effect of these agents cannot be disentangled, limiting the ability to attribute observed changes specifically to CHX.
4. The limited or absent microbiological effect observed probably reflects the restricted penetration of mouthwashes into deep peri-implant pockets. This issue should be considered when evaluating antiseptic rinses as treatment modalities.
5. Factors such as smoking habits, systemic conditions (e.g., epilepsy in one participant), and oral hygiene practices may have influenced microbiota composition, but their impact could not be fully assessed due to the small cohort size.

## 5. Materials and Methods

### 5.1. Subject Recruitment

Twenty-two patients with PI were recruited from the University Dental Clinic of the Faculty of Medicine and Dentistry in the Universidade de Santiago de Compostela (Spain). Selected patients were diagnosed with PI following the guidelines proposed by the eighth European Workshop on Periodontology and the 2017 World Workshop on the Classification of periodontal and peri-implant diseases and conditions: presence of BOP and/or suppuration and marginal bone loss (MBL) (≥3 mm) [3,4,66]. Marginal bone loss was assessed radiographically. Vestibular and lingual probing depth was measured at 3 points of the PI pocket with a CP-11 Hu-Friedy periodontal probe.

All patients received their implants at least a year before the study and presented symptoms of PI for more than one month. None of the patients were treated with CHX or antibiotics for fifteen days or three months, respectively, before the sampling procedure. No other exclusion criteria were applied. Patients were divided into the 0.05% CHX group (Perio-Aid^®^ maintenance, Dentaid, Cerdanyola del Vallès, Spain) or the 0.12% CHX group (Perio-Aid^®^ treatment, Dentaid, Spain). They were instructed to use the mouthwash (15 mL) three times a day for 30 s after their routine toothbrushing. This intensive treatment was prescribed in order to maximize the effects of the treatments [67]. Patients were also instructed not to use any other antiseptic or cleaning procedure during the treatment period (15 days). All patients signed a written informed consent approved by the Comité de Ética de la Investigación con medicamentos de Galicia (protocol 2018/560, extended by protocol 2020/007).

### 5.2. Sample Collection and Preparation

All samples were obtained by the same odontologist (F. Pose-Otero), and in all cases, the sampling procedure was supervised by at least one experienced odontologist from the research team. No mechanical debridement was performed before sampling, with the treatment being exclusively chemical. The surfaces of crowns were cleaned with a scaler before sampling to avoid the contamination of the paper points with supragingival biofilm. The sampling sites were isolated using dental cotton rolls to avoid saliva contamination. The subgingival biofilm samples for microbiological analyses were collected by insertion of paper points into the PI pocket before and after a 15-day CHX treatment. Four paper points were inserted in the deepest portion of the PI pocket for 30 s, since a previous study demonstrated that this method allowed the recovery of a higher number of bacterial genera than the use of curettes [68]. Samples were stored in the first reagent of the “DNeasy PowerBiofilm Kit” (Qiagen, Germantown, MD, USA) at −20 °C for no longer than 45 days before being extracted. The study was performed between January and February 2020.

### 5.3. Microbial DNA Extraction

Genomic DNA for metagenomic analysis was extracted using “DNeasy PowerBiofilm Kit” (Qiagen, Germantown, MD, USA) following the manufacturer’s instructions. Briefly, biofilms were subjected to chemical and mechanical lysis. Proteins and inhibitors were removed, followed by pH-driven precipitation of large insoluble macromolecules. Total genomic DNA was captured using a silica spin filter column and finally eluted. DNA concentration was measured using a NanoDrop (Thermo Scientific, Waltham, MA, USA).

### 5.4. Library Preparation

Microbial genomic DNA (5 ng/µL in 10 mM Tris, pH 8.5) was used to amplify the 16S rRNA gene V3 and V4 regions. After size verification, the library was sequenced using a 2 × 300 pb paired-end run (MiSeq Reagent kit V3(MS-102-3001)) on a MiSeq Sequencer according to the manufacturer’s instructions (Illumina, San Diego, CA, USA).

### 5.5. Bioinformatics and Microbial Diversity Analysis

Quality assessment and denoising were performed using the prinseq-lite program [69]. The pipeline DADA2 [70] was used to analyze and cluster the sequences into amplicon sequence variants, classified to the genus level with the SILVA database [71]. Computations and statistics were carried out in R Statistics 4.1.3 [72] using knitr, knitcitations, markdown [73,74,75], biostrings, and vegan [76]. The differential relative abundance of the bacterial genera identified was assessed with the packages Phyloseq 1.38 and DESeq2 1.34 [77]. Differences were filtered with a log2 fold change threshold higher than 2 and by their base mean value, discarding the lower quartile. Beta diversity was studied using a PCoA of weighted UniFrac distances using the package Phyloseq. Differences between samples, grouped according to the studied variables, were analyzed using the PERMANOVA test implemented in the adonis function of the package vegan 2.6-2 [76]. Alpha diversity was analyzed with the R package Phyloseq 1.30 [78], using the Chao1 and the Shannon and Simpson indices as richness and diversity estimators. The data’s normality and homoscedasticity were assessed before choosing parametric or nonparametric tests included in the package stat 0.1 [71]. Student’s *t*-test or Wilcoxon tests were applied to compare samples depending on the result of normality and homoscedasticity tests.

## 6. Conclusions

1. Even though the study of a larger number of patients is required in order to withdraw solid conclusions, this pilot study indicates that short-time, intensive prescription of CHX mouthwashes should be recommended with caution for the treatment of PI, since it does not have any statistically significant effect on the lesion-associated microbiota, with a large variability in the patients’ response, and may have undesirable side effects.
2. The lowest CHX concentration (0.05%) should be considered for combined or long-term therapies, since no statistically significant differences were observed in comparison with 0.12% CHX.

## Figures and Tables

**Table 1:** Average sex and age of the 0.05% and 0.12% chlorhexidine (CHX) groups. Other clinical parameters, implant characteristics, and risk factors are shown in Supplementary Tables S1 and S2.

	0.05% CHX Group	0.12% CHX Group
**Age (years)**	65 ± 7.4	65 ± 12.6
**Sex, *n* (%)**		
Female	6 (54.54%)	6 (54.54%)
Male	5 (45.45%)	5 (45.45%)
**Average implant age before PI onset (years)**	3.13	3.5

## Data Availability

The original data presented in the study are openly available in Bioproject at https://www.ncbi.nlm.nih.gov/bioproject/PRJNA1244316, accessed on 17 September 2025. A previous version of this work was published as a preprint on the Research Square platform (https://doi.org/10.21203/rs.3.rs-4217658/v1).

## Supplementary Materials

The following supporting information can be downloaded at: https://www.mdpi.com/article/10.3390/antibiotics14101032/s1. Figure S1: Richness and diversity and Principal Coordinates Analysis of weighted Unifrac plots of the microbiome structure of the initial samples—baseline—of patients receiving treatments with CHX 0.05% and CHX 0.12%; Table S1: List of comparisons of the alpha diversity measures between the different groups of this study and the p-values obtained in each comparison; Table S2: List of comparisons of the beta diversity measures between the different groups of this study and the p-values obtained in each comparison; Table S3: Significant differential abundance of bacterial genus, sorted by their significance level in each comparison; Table S4: Clinical information of patients treated with 0.05% CHX; Table S5: Clinical information of patients treated with 0.12% CHX.
