# Supplementary material for: The Effect of Chlorhexidine Mouthwashes on the Microbiota Associated with Peri-Implantitis Lesions: A Pilot Study
Source: Antibiotics (Basel). 2025 Oct 15;14(10):1032. doi: 10.3390/antibiotics14101032 (PMC12561514; doi:10.3390/antibiotics14101032)
Supplement: Supplementary file 1 [file antibiotics-14-01032-s001.zip › antibiotics-3910914-supplementary.pdf]

Supplementary Information

The Effect of Chlorhexidine Mouthwashes on the Microbiota Associated with Peri-Implantitis Lesions: A Pilot Study

Félix Pose-Otero [1], Alexandre Arredondo [2], Ana Parga [3,4], Andrea Muras [3,†], Mercedes Gallas [1], Paz Otero-Casal [1,5], José Manuel Pose-Rodríguez [1] and Ana Otero [3,4,*]

[1] Departamento de Cirurxía e Especialidade Médico-Cirúrxica, Facultade de Medicina e Odontoloxía, Universidade de Santiago de Compostela, 15705 Santiago de Compostela, Spain; felixramon.pose@rai.usc.es (F.P.-O.); mercedes.gallas.torreira@usc.es (M.G.); paz.otero.casal@sergas.es (P.O.-C.); josemanuel.pose@usc.gal (J.M.P.-R.)

[2] Department of Microbiology, Dentaid Research Center, 08290 Cerdanyola Del Vallès, Spain; alex.arredondo@dentaid.es

[3] Department of Microbiology and Parasitology, Faculty of Biology, , Universidade de Santiago de Compostela, 15705 Santiago de Compostela, Spain; ana.parga.martinez@usc.es (A.P.); andrea.muras.mora@sergas.es (A.M.)

[4] Aquatic One Health Research Center (ARCUS), Universidade de Santiago de Compostela, 15705 Santiago de Compostela, Spain

[5] Unit of Oral Health, C.S. Santa Comba-Negreira, Servicio Galego de Saúde (SERGAS), 15830 Negreira, Spain

* Correspondence: anamaria.otero@usc.es; Tel.: +34-8818-16913

[†] Present address: Servicio de Microbiología, Instituto de Investigación Biomédica A Coruña (INIBIC), Complexo Hospitalario Universitario A Coruña, Universidade da Coruña (UDC), 15008 A Coruña, Spain

Supplementary information

Table S1 Clinical information of patients treated with 0.05% CHX. nd: not determined

	Age	Sex	Smoker	Medical History	Medical Treatments	Last antibiotic treatment	Last CHX treatment	Use of dental irrigator	Implant position	Type of connection	Implant brand	Unitary	Implant placement date	PI symptom onset date	Bleeding on probing	Probing depth (mm)
P1	65	F	No	Asthma	Symbiconin 16 mg	05/2018	11/11/19	Yes	24	Internal hexagon	Biohorizons	No	14/06/14	28/03/16	Yes	V 3,4,6<br>L 3,4,6
P2	57	F	No	nd	No	27/11/14	23/03/17	No	45	Internal hexagon	Biohorizons	Yes	nd	nd	Yes	V 4,2,4<br>L 2,4,2
P3	57	M	No	nd	nd	18/02/19	04/03/19	No	13	Internal hexagon	Biohorizons	No	13/04/12	21/12/15	Yes	V 8,3,4<br>L 4,3,2
P4	64	M	No	Cholesterol	Basticina cloxatan	12/09/16	12/06/17	No	14	Internal hexagon	Biohorizons	Yes	06/02/14	14/03/16	Yes	V 3,3,4<br>L 3,3,2
P5	66	F	No	nd	nd	20/11/17	22/07/19	No	24	Internal hexagon	Biohorizons	No	19/03/14	20/11/17	No	V 4,1,5<br>L 3,3,2
P6	70	F	No	Depression<br>Tension<br>Stomach	Alprazolan<br>Fentanilo matrix<br>Valsatan<br>Omeprazol	nd	A month before fist sampling	Yes	45	Internal hexagon	Biohorizons	Yes	14/11/16	nd	No	V 4,1,5<br>L 3,4,5
P7	58	M	No	nd	nd	03/10/19	03/10/19	No	15	Internal octagon	Strauman	No	12/05/09	20/04/15	No	V 5,3,6<br>L 8,3,3
P8	62	F	Yes	nd	nd	23/02/15	14/12/18	Yes	44	Internal hexagon	Biohorizons	Yes	16/12/10	12/03/15	No	V 3,1,4<br>L 3,7,4
P9	62	M	No	Epilepsy	Fentoina	Cefalosporine six months before fist sampling	A month and a half before fist sampling	No	44	Internal hexagon	Biohorizons	No	nd	nd	No	V 3,1,3<br>L 1,1,1
P10	82	M	No	nd	nd	23/04/18	nd	No	35	Internal hexagon	Biohorizons	No	27/05/13	25/04/16	No	V 2,3,3<br>L 2,1,3
P11	72	F	No	nd	nd	23/04/18	23/12/19	No	46	Internal hexagon	Biohorizons	No	21/10/15	10/10/16	No	V 7,6,6<br>L 6,4,8

Supplementary information

Table S2 Clinical information of patients treated with 0.12% CHX. nd: not determined

	Age	Sex	Smoker	Medical History	Medical Treatments	Last antibiotic treatment	Last CHX treatment	Use of dental irrigator	Implant position	Type of connection	Implant brand	Unitary	Implant placement date	PI symptom onset date	Bleeding on probing	Probing depth (mm)
P1	65	F	No	nd	nd	08/09/16	nd	No	33	Internal hexagon	Biohorizons	Yes	09/06/16	23/03/18	nd	V 4,1,2<br>L 4,3,3
P2	76	F	No	nd	Tension cholesterol	2 months before fist sampling	23/12/19	No	44	Internal octagon	Straumann	No	05/03/09	11/11/19	No	V 4,1,2<br>L 4,2,2
P3	72	M	No	nd	nd	05/11/18	10/10/19	No	13	Internal hexagon	Gt medical	No	09/11/18	04/07/19	Yes	V 6,6,6<br>L 6,6,6
P4	84	M	No	nds	Simtron prostate	08/02/16	A year before fist sampling	No	14	Internal hexagon	Biohorizons	No	nd	nd	No	V 6,4,5<br>L 7,7,6
P5	77	M	No	nd	nd	2 months ago	04/07/19	No	24	Internal hexagon	Biohorizons	No	nd	nd	No	V 4,4,5<br>L 6,7,1
P6	55	F	No	nd	nd	02/11/17	nd	Yes	26	Internal hexagon	Biohorizons	No	16/07/14	25/02/16	nd	V 2,2,4<br>L 2,2,6
P7	57	F	No	nd	nd	25/02/17	15/03/18	No	46	Internal hexagon	Biohorizons	Yes	20/02/17	05/11/18	No	V 5,2,3<br>L 3,2,2
P8	52	M	No	nd	nd	More than year before fist sampling	A year before fist sampling	Yes	17	Internal octagon	Straumann	No	08/03/13	nd	No	V 6,3,3<br>L 6,3,3
P9	75	M	No	HTA cholesterol	HTA Cholesterol Dilutol ramipil	More than year before fist sampling	nd	Yes	13	Internal octagon	Gt medical	No	03/08/11	nd	nd	V 6,4,3<br>L 5,5,6
P10	58	F	No	nd	nd	More than six months before fist sampling	A month before fist sampling	Yes	16	Internal hexagon	Biohorizons	No	02/03/11	08/03/16	Yes	V 4,6,8<br>L 3,3,6
P11	44	F	Yes	nd	Antidepressant eutirox	More than six months before fist sampling	More than six months before fist sampling	No	35	Internal hexagon	Biohorizons	No	08/10/14	nd	No	V 6,6,6<br>L 3,3,6

Supplementary information

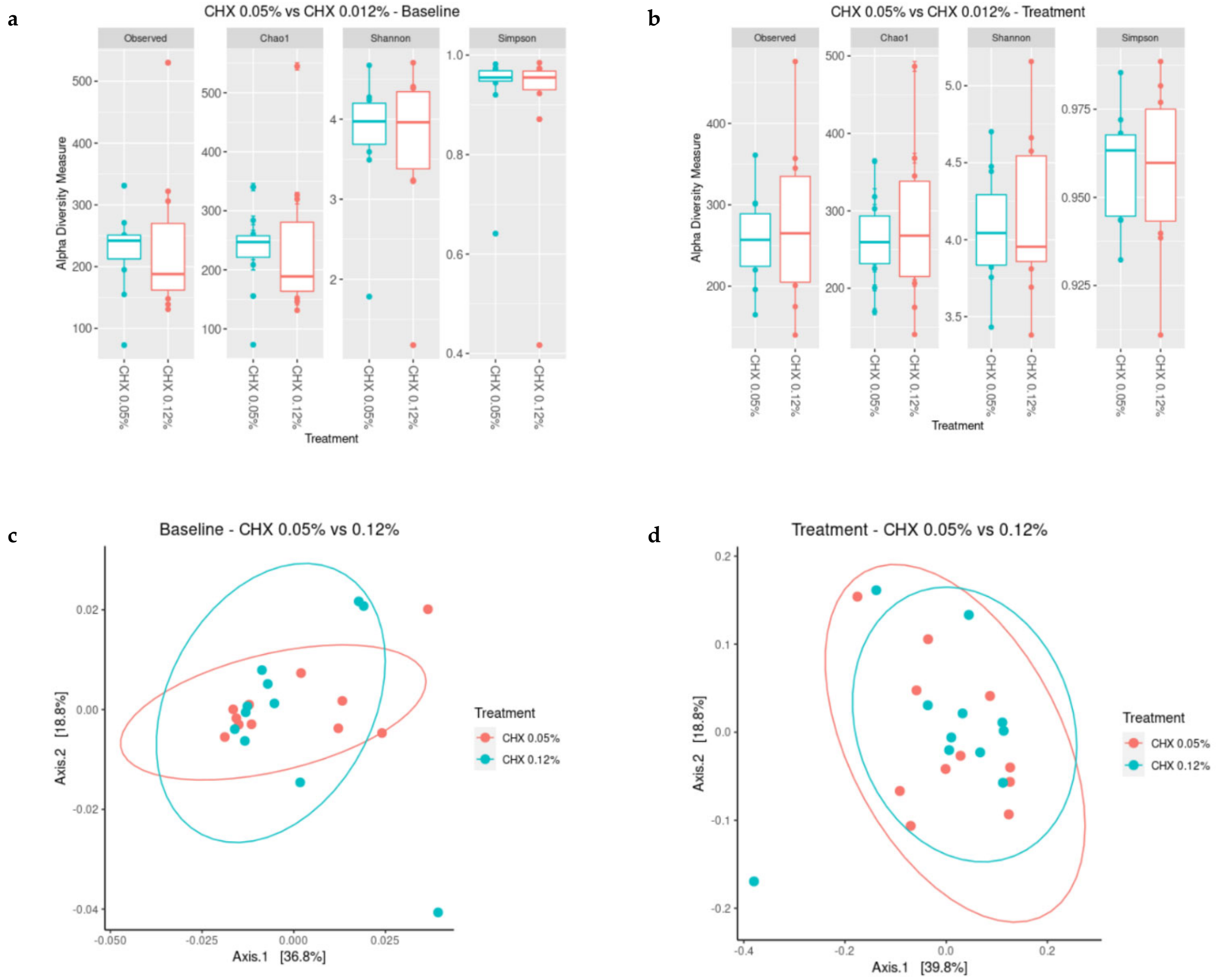

Figure S1 Richness and diversity (a and b) and Principal Coordinates Analysis of weighted Unifrac plots (c and d) of the microbiome structure of the initial samples -baseline- of patients receiving treatments with CHX 0.05% and CHX 0.12% (a and c) and the same comparison after 15 days of treatment (b and d)

Table S3 List of comparisons of the alpha diversity measures between the different groups of this study and the p values obtained in each comparison. Bsln: Baseline, Ttm: Treatment

Comparison	Observed	Chao1	Shannon	Simpson
0.05 - Bsln vs Ttm	0.3505	0.3983	0.3983	0.1372
0.12 - Bsln vs Ttm	0.2664	0.3066	0.3066	0.3983
Bsln - 0.05 vs 0.12	1	0.9645	0.8939	0.8939
Ttm - 0.05 vs 0.12	0.5049	0.5049	0.4498	0.9645

Table S4 List of comparisons of the beta diversity measures between the different groups of this study and the p values obtained in each comparison. Bsln: Baseline, Ttm: Treatment

Comparison	R2	p-value	Betadisper
0.05% Bsln vs Ttm	0.01756	0.967	0.205
0.12% Bsln vs Ttm	0.01765	0.971	0.398
Bsln 0.05 vs 0.12	0.01531	0.995	0.626
Ttm 0.05 vs 0.12	0.02688	0.825	0.51

Table S5 Significant differential abundance of bacterial genus, sorted by their significance level in each comparison

**Baseline vs Treatment 0.05% CHX**			
**Genus**	**baseMean**	**log2FoldChange**	**padj**
Jonquetella	6.821091662	3.660473942	0.041372206
**Baseline vs Treatment 0.12% CHX**			
**Genus**	**baseMean**	**log2FoldChange**	**padj**
Cardiobacterium	16.27063825	-3.301896303	0.02198203
Actinobacillus	4.884124608	-3.099539268	0.02198203
Blautia	5.656159263	3.17402149	0.02198203
HT002	5.10903941	3.006760632	0.02198203
Suttonella	3.687251189	2.464875265	0.040303159
[Eubacterium] yurii group	4.056025539	-2.636230653	0.048046152
